# Clinical study of falls among inpatients with hematological diseases and exploration of risk prediction models

**DOI:** 10.3389/fpubh.2023.1150333

**Published:** 2023-06-27

**Authors:** Jing Wang, Bin Chen, Fang Xu, Qin Chen, Jing Yue, Jingjing Wen, Fang Zhao, Min Gou, Ya Zhang

**Affiliations:** Department of Hematology, Mianyang Central Hospital, School of Medicine, University of Electronic Science and Technology of China, Mianyang, China

**Keywords:** hematology, inpatient, falls, risk prediction, risk factor

## Abstract

**Background:**

Falls are serious health events that can cause life-threatening injuries, especially among specific populations. This study assessed the risk factors associated with falls among inpatients with hematological diseases and explored the predictive value of fall risk assessment models.

**Methods:**

Clinical data from 275 eligible hematology disease patients who visited Mianyang Central Hospital with or without falls from September 2019 to August 2022 were retrospectively analyzed. Fall risk scores were determined in all included patients. Clinical characteristics were compared between patients with and without falls. Binary logistic regression models were used to screen for potential fall-specific risk factors among hospitalized patients with hematology diseases.

**Results:**

Falls occurred in 79 cases. Patients in the fall group had a higher Charlson Comorbidity Index (CCI), a higher incidence of diabetes mellitus, visual impairment, hematological malignancies, and maintenance of stable disease stage, higher glucose levels, and a greater proportion of dizziness, nocturnal defecation, and receipt of intensive chemotherapy than those in the non-fall group (all *P* < 0.05). Fall patients were also more likely to have used diuretics, laxatives, sedative-sleeping drugs, analgesics, albumin, and calcium, and to have had catheters placed. The Barthel Index, grade of nursing care, support of chaperones, body temperature, nutrition score, and pain score also differed significantly between the two groups (all *P* < 0.05). Multivariable logistic regression analysis showed that the maintenance of stable disease stage (OR = 4.40, 95% CI 2.11–9.18, *P* < 0.001), use of sedative and sleeping drugs (OR = 4.84, 95% CI 1.09–21.49, *P* = 0.038), use of diuretics (OR = 5.23, 95% CI 2.40–11.41, *P* < 0.001), and intensive chemotherapy (OR = 10.41, 95% CI 3.11–34.87, *P* < 0.001) were independent risk factors for falls. A high Barthel Index (OR = 0.95, 95% CI 0.93–0.97, *P* < 0.001), a high level of nursing care (OR = 0.19, 95% CI 0.04–0.98, *P* = 0.047), and availability of family accompaniment (OR = 0.15, 95% CI 0.06–0.34, *P* < 0.001) were protective factors for falls. A ROC curve analysis was used to evaluate the predictive value of different fall-specific risk scales among inpatients with hematological diseases. The Johns Hopkins Fall Risk Rating Scale had high sensibility and specificity with an area under the curve of 0.73 (95% CI 0.66–0.80, *P* < 0.001).

**Conclusion:**

The Johns Hopkins Fall Risk Scale had a strong predictive value for falls among hospitalized patients with hematology diseases and can be recommended as a valid tool for clinical use.

## 1. Introduction

Falls are serious public health events that occur in both the community and hospitals and can lead to life-threatening injuries. They are not only associated with physical and mental pain but also prolong the length of hospital stay, increase the cost of hospitalization, consume medical resources, increase the workload of medical staff, reduce patient and medical staff satisfaction, and can become a source of medical disputes (1, 2). It is estimated that over 84% of all adverse events in hospitalized patients are related to falls (3). The incidence of falls is an important indicator of the quality of healthcare and patient safety (4, 5). The incidence and injury rates of falls vary by geographic region and population. In foreign countries, the fall rate ranges from 2.6 to 7% and the injury rate ranges from 23 to 42% (6). In 2017, the fall rate in China was 0.054%, and the injury rate was 73.68% (7). Evidence indicates that fall rates are higher in special populations including older adults and those with specific diseases (8, 9).

Falls in patients with hematological diseases may lead to more complicated and serious adverse consequences or even death due to a higher risk of complications such as thrombocytopenia, abnormal coagulation function, and bone disease (10). In addition, chemotherapy is the primary treatment for most hematological patients, and the incidence of falls in chemotherapy patients is reported to be as high as 22–50% (11–13). Most studies on falls have focused on the economic implications and older adult populations (14, 15). Thus, there is a need to better understand fall risk among hospitalized patients with hematologic diseases. In addition, while a few studies have assessed the risk prediction of falls, particular risk factors for falls among inpatients with hematologic diseases remain less well reported. This study investigated fall-related risk factors and sought to develop a fall prediction model among inpatients with hematologic diseases.

## 2. Materials and methods

### 2.1. Study population

This retrospective study included 79 patients with non-syncopal falls and 196 patients without falls who were hospitalized in the Department of Hematology at Mianyang Central Hospital (Mianyang, China) from September 2019 to August 2022. Patients who were ≥15 years of age, had a hematologic-related disease, could read and communicate normally, and were willing to participate were included in the study. Those with mental illness or cognitive impairment, poor compliance, or other malignant diseases, who were reported missing important data or were unavailable, and who had a fall occurring outside the hospital or a hospital stay of < 1 day were excluded.

### 2.2. Clinical information

Basic clinical information and relevant factors associated with falls were collected for all patients, including general characteristics (gender, age, weight, and body mass index), social characteristics (educational background and accompanying status), physiological status (temperature, blood pressure, nutrition, pain, Barthel Index, vision, and hearing), disease status (Charlson's co-morbidity index (CCI), comorbidities, type of hematological disease, stage of disease, history of oncologic disease, and history of falls), and type of medical support (level of care, use of a walking aid, infusion pumps, placement of catheters, concomitant chemotherapy, the intensity of chemotherapy, medication, and laboratory indicators) (16–21). The patients were divided into a fall group and a non-fall group depending on whether they had experienced a fall.

### 2.3. Fall risk assessment scales

The Hendrich Fall Risk Assessment Scale, Thomas Fall Risk Assessment Tool, Morse Fall Assessment Scale, Johns Hopkins Fall Risk Assessment Scale, Falls Risk Assessment Tool (FRAT), and Fall Risk Assessment Tool for older adults (22–28) were used to assess fall risk. All patients were assessed for the first time within 2 days after admission and individuals in the fall group were assessed after their fall.

### 2.4. Fall definition

A fall was defined according to the International Classification of Diseases (ICD) standard code 10th edition ICD-10 falls category (W00–W19) (29).

### 2.5. Statistical analysis

Statistical analysis was conducted using SPSS software (IBM Corp, Released 2019, IBM SPSS Statistics for Windows, version 25.0, Armonk, NY, USA). Categorical variables were expressed as rates, continuous variables were expressed as means ± standard deviations, and the measures of a skewed distribution were expressed as median and rank mean. Univariate analysis was conducted first, the χ^2^ test was used for categorical variables, and the Kolmogorov–Smirnov test was used for measurement data. Measurements that conformed to a normal distribution were tested using an independent sample *t*-test, while non-normal distributions were assessed using a rank sum test. Binary logistic regression models were used for the analysis of multiple factors. All variables that were identified as potentially meaningful factors by univariate analysis (*P* < 0.05) were included as covariates in the binary logistic regression analysis. These included the Charlson Comorbidity Index (CCI), oncological disease, type of hematological disease, stage of disease, body temperature, level of care, Barthel Index, nutrition score, pain measurement, blood glucose, diabetes history, visual impairment, dizziness, nocturnal urination and defecation, placement of catheters, chemotherapy intensity, application of diuretics, laxatives, sedative-hypnotics, and analgesics, and lack of chaperone support. Receiver operating characteristic (ROC) curves were plotted according to the sensitivity and specificity of RAMs, and the area under the curve (AUC) and 95% confidence interval (CI) were also calculated. A *P*-value of < 0.05 was considered statistically significant.

### 2.6. Ethical approval

The study was approved by the Ethics Committee at Mianyang Central Hospital (approval number: S20220224-01). The procedures used in this study adhered to the tenets of the Declaration of Helsinki.

## 3. Results

### 3.1. Clinical characteristics of hospitalized patients with hematological diseases in the fall and non-fall groups

Of the 275 patients, 79 (28.73%) patients had experienced falls. The fall group had a higher median Charlson Comorbidity Index (CCI) (4 vs. 3, *P* = 0.001), a higher incidence of diabetes mellitus (19.00 vs. 8.70%, *P* = 0.016), greater visual impairment (11.40 vs. 2.60%, *P* = 0.007), more hematological malignancies (81.00 vs. 60.20%, *P* = 0.001), a higher proportion of patients with stable disease stage (57.00 vs. 28.10%, *P* < 0.001), higher glucose levels (7.2 vs. 5.9 mmol/L, *P* < 0.001), more dizziness (79.70 vs. 55.60%, *P* < 0.001), and more symptoms of nocturnal defecation (41.80 vs. 11.70%, *P* < 0.001). Patients in the fall group were more likely to have received chemotherapy (53.20 vs. 27.60%, *P* < 0.001), intensive chemotherapy regimens vs. standard or reduced intensity regimens (40.50 vs. 31.00 vs. 28.50%, *P* = 0.026), diuretics (49.40 vs. 12.80%, *P* < 0.001), laxatives (13.90 vs. 4.60%, *P* = 0.007), sedative-hypnotics (12.70 vs. 2.00%, *P* = 0.001), analgesics (25.30 vs. 11.20%, *P* = 0.003), and catheter placement (54.40 vs. 28.10%, *P* < 0.001). These patients also had lower albumin levels (34.3 ± 7.2 vs. 39.0 ± 7.3, *P* < 0.001), calcium levels (2.1 vs. 2.2, *P* < 0.001), a lower Barthel Index (70 vs. 80, *P* < 0.001), a lower level of nursing care (87.30 vs. 98.50%, *P* < 0.001), less family accompaniment (60.80 vs. 83.70%, *P* < 0.001), lower body temperature (Z = −2.2, *P* = 0.026), a lower nutrition score (Z = −5.2, *P* < 0.001), and higher pain measurement (Z = −3.1, *P* = 0.002) than those in the non-fall group (Table 1).

**Table 1:** Clinical features of hospitalized patients from hematology departments with and without falls.

**Variables**	**Fallers**	**Non-fallers**	**x^2^ /T/Z**	***P*-value**
**Admissions**, ***n***	79	196		
**General characteristics of patients**				
Gender (Male/Female), *n*	45/34	108/88	0.1	0.779
Age (mean rank)	150.8	132.8	−1.7	0.089
Weight (mean rank)	133.4	137.7	–0.4	0.679
BMI, kg/m^2^	22.2 ± 3.4	22.4 ± 3.2	−0.5	0.634
**Social characteristics of patients**				
**Education level**, ***n*** **(%)**				
Junior high school and below	56 (70.90)	160 (81.60)	5.3	0.071
High school	12 (15.20)	24 (12.20)		
University and above	11 (13.90)	12 (6.10)		
**With chaperones**, ***n*** **(%)**	48 (60.80)	164 (83.70)	16.7	< 0.001^*^
**Physiological status of patients**				
Median body temperature (range) °C	36.6 (36.0–39.8)	36.5 (36.0–40.0)	−2.2	0.026^*^
**Blood pressure, mmHg**				
Systolic blood pressure (mean rank)	142.2	136.3	−0.6	0.6581
Diastolic blood pressure (mean rank)	136.2	138.7	−0.2	0.812
Median nutrition score (range)	3 (0–6)	2 (0–6)	−5.2	< 0.001^*^
Pain measurement (mean rank)	155.7	130.9	−3.1	0.002^*^
Median Barthel Index (range)	70 (0–100)	80 (15–100)	−4.8	< 0.001^*^
**Organ function of patients**, ***n*** **(%)**				
Visual impairment	9 (11.40)	5 (2.60)	7.4	0.007^*^
Hearing impairment	6 (7.60)	8 (4.10)	0.8	0.370
**Concomitant/combined disease status**				
Median CCI (range)	4 (0–12)	3 (0–9)	−4.3	0.001^*^
**Comorbidities**, ***n*** **(%)**				
Diabetes mellitus	15 (19.00)	17 (8.70)	5.8	0.016^*^
Hypertension	15 (19.00)	30 (15.40)	0.5	0.466
Dizziness	63 (79.70)	109 (55.60)	14.0	< 0.001^*^
Increased nocturia or defecation	33 (41.80)	23 (11.70)	31.3	< 0.001^*^
**Type of hematologic disease**, ***n*** **(%)**				
Leukemia	35 (44.30)	62 (31.60)	11.1	0.011^*^
Malignant lymphoma	20 (25.30)	37 (18.90)		
Multiple myeloma	9 (11.40)	19 (9.70)		
Nonmalignant diseases	15 (19.00)	78 (39.80)		
**Disease stage**, ***n*** **(%)**				
Relapsing or refractory phase	34 (43.00)	141 (71.90)	20.3	< 0.001^*^
Stable stage	45 (57.00)	55 (28.10)		
**Malignancies**, ***n*** **(%)**	64 (81.00)	118 (60.20)	10.9	0.001^*^
**Fall history**, ***n*** **(%)**	10 (12.70)	14 (7.10)	2.2	0.143
**Patient consultation status**				
**Level of nursing care**, ***n*** **(%)**				
Grade I Care	69 (87.30)	193 (98.50)	13.1	< 0.001^*^
Grade II Care	10 (12.70)	3 (1.50)		
Use of a walking aid, *n* (%)	12 (15.20)	26 (13.30)	0.2	0.676
Use of infusion pump, *n* (%)	21 (26.60)	33 (16.80)	3.4	0.066
Catheter placement, *n* (%)	43 (54.40)	55 (28.10)	17.1	< 0.001^*^
**Receiving chemotherapy**, ***n*** **(%)**				
Intensive program	17 (21.50)	10 (5.10)	25.7	< 0.001^*^
Standard program	13 (16.50)	31 (15.80)		
Dose reduction program	12 (15.20)	13 (6.60)		
No chemotherapy	37 (46.80)	142 (72.40)		
**Medication use**, ***n*** **(%)**				
Diuretics	39 (49.40)	25 (12.80)	42.3	< 0.001^*^
Laxatives	11 (13.90)	9 (4.60)	7.3	0.007^*^
Sedative sleeping pills	10 (12.70)	4 (2.00)	11.0	0.001^*^
Analgesics	20 (25.30)	22 (11.20)	8.6	0.003^*^
Antihypertensive drugs	7 (8.90)	15 (7.70)	0.1	0.738
**Laboratory metrics**				
**Blood count**				
RBC count, × 10^12^ /L	2.8 ± 1.1	3.0 ± 1.2	−1.8	0.071
Hemoglobin (mean rank), g/L	124.6	142.7	−1.7	0.086
Platelet (mean rank), × 10^9^ /L	125.5	142.4	−1.6	0.109
**Biochemical index**				
Albumin, g/L	34.3 ± 7.2	39.0 ± 7.3	−4.8	< 0.001^*^
Potassium, mmol/L	3.8 ± 0.8	3.9 ± 0.5	−0.4	0.686
Median calcium (range), mmol/L	2.1 (1.5–2.5)	2.2 (1.7–3.3)	−4.3	< 0.001^*^
Median glucose (range), mmol/L	7.2 (3.4–17.5)	5.9 (3.4–26.6)	−4.0	< 0.001^*^

BMI, Body mass index; CCI, Charlson Comorbidity Index; RBC, Red blood cell. ^*^Statistically significant.

### 3.2. Analysis of the clinical characteristics of hospitalized patients with hematological diseases in the fall group

Of the 79 fall events, most occurred during day shifts (35.40%) and in the bathroom (41.80%). The median time of fall occurrence was 8 days after admission. Analysis of the surrounding environment showed that the floor was normal and brightly lit for 93.70 and 64.60% of events, respectively. Most fall patients (68.40%) had handrails and 68.40% had no pager in the surrounding area. Social factor analysis of on-duty nurses showed that the median length of service was 5 years, 83.50% had a bachelor's degree or higher, 96.20% had a junior title, 65.80% were married, and 60.80% had given birth. Most patients with falls (62.00%) did not suffer obvious trauma and most falls (97.50%) were grade III nursing adverse events (Table 2). Univariate analysis showed that falls were more associated with the physiological status, disease status, and medical support of patients than their general characteristics.

**Table 2:** Clinical characteristics of falls occurring in patients hospitalized with hematological diseases.

**Variables**	**Falling**
**Admissions**, ***n***	79
**Event status**	
**Number of shifts**, ***n*** **(%)**	
Day shift	28 (35.40)
On the night	23 (29.10)
Next night	28 (25.40)
**Location** ***n*** **(%)**	
Bedside	22 (27.80)
Restrooms	33 (41.80)
Other	24 (30.40)
**Median Length of admission** (range)	8 (1–55)
**Environmental factors**	
**Ground**, ***n*** **(%)**	
Wet and slippery	2 (2.50)
unevenness	3 (3.80)
No special	84 (93.70)
**Light**, ***n*** **(%)**	
Bright	51 (64.60)
Dim	28 (35.40)
**With handrails**, ***n*** **(%)**	54 (68.40)
**Pager**, ***n*** **(%)**	
None	54 (68.40)
Inconvenience	8 (11.40)
Convenient	16 (20.30)
**Event ending**	
**Injury degree**, ***n*** **(%)**	
None	49 (62.00)
Minor injuries	28 (35.40)
Serious injuries	2 (2.50)
**Nursing adverse event level**, ***n*** **(%)**	
Grade II	2 (2.50)
Grade III	77 (97.50)
**Status of nurses on duty**	
**Median length of service** (rang)	5 (1–28)
**Academic qualifications**, ***n*** **(%)**	
Bachelor's degree and above	66 (83.50)
Specialized and below	13 (16.50)
**Title**, ***n*** **(%)**	
Intermediate and above	3 (3.80)
Below intermediate	76 (96.20)
**Marriage**, ***n*** **(%)**	
Married	52 (65.80)
Unmarried	27 (34.20)
**Maternity**, ***n*** **(%)**	
None	29 (36.70)
Pregnancy	2 (2.50)
Childbirth	48 (60.80)

### 3.3. Multivariate analysis of fall-related risk factors among inpatients with hematological diseases

Univariate analysis found that the Charlson Comorbidity Index (CCI), oncological disease, hematological disease type, disease state, body temperature, level of care, Barthel Index, nutrition score, pain measurement, blood glucose, diabetes history, visual impairment, dizziness, increased nocturnal urination and defecation, placement of catheters, the intensity of chemotherapy, the application of diuretics, laxatives, sedative-hypnotics, and analgesics, and the absence of chaperone support were possible risk factors for falls. These variables were analyzed by multivariable logistic regression using the input method with the inclusion criterion of *P* < 0.05 and the exclusion criterion of *P* > 0.1.

Stable stage disease (OR = 4.40, 95% CI 2.11–9.18, *P* < 0.001), the use of sedatives or sleeping medication (OR = 4.84, 95% CI 1.09–21.49, *P* = 0.038), the use of diuretics (OR = 5.23, 95% CI 2.40–11.41, *P* < 0.001), and intensive chemotherapy regimens (OR = 10.41, 95% CI 3.11–34.87, *P* < 0.001) were independent risk factors for falls. A higher Barthel Index (OR = 0.95, 95% CI 0.93–0.97, *P* < 0.001), a higher level of nursing care (OR = 0.19, 95% CI 0.04–0.98, *P* = 0.047), and family accompaniment (OR = 0.15, 95% CI 0.06–0.34, *P* < 0.001) were protective factors for falls (Table 3).

**Table 3:** Risk Factors associated with falling (multivariate logistic regression analysis).

**Variables**	**OR**	**95% CI**	***P*-value**
Level I care	0.19	0.04–0.98	0.047
Barthel index	0.95	0.0.93–0.97	< 0.001
With chaperones	0.15	0.06–0.34	< 0.001
Diuretics	5.23	2.40–11.41	< 0.001
Sedative sleeping pills	4.84	1.09–21.49	0.038
Stabilization stage	4.40	2.11–9.18	< 0.001
Receiving chemotherapy			0.002
Intensive programs	10.41	3.11–34.87	< 0.001
Standard program	1.50	0.58–3.85	0.400
Reduction programs	2.48	0.74–8.31	0.141

CCI, Charlson Comorbidity Index. Variables that had a *P*-value < 0.05 (CCI, Malignancies, Type of hematologic disease, Disease stage, Body temperature, Level of care, Barthel Index, Nutrition score, Pain score, Glucose, Diabetes mellitus, Visual impairment, Dizziness, Increased nocturia or defecation, Catheter placement, Receiving chemotherapy, Diuretics, Laxatives, Sedative sleeping pills, Analgesics, With chaperones) in univariable analysis were selected for inclusion in the multivariable model.

### 3.4. Predictive capability of fall risk assessment scales

**Figure 1:**
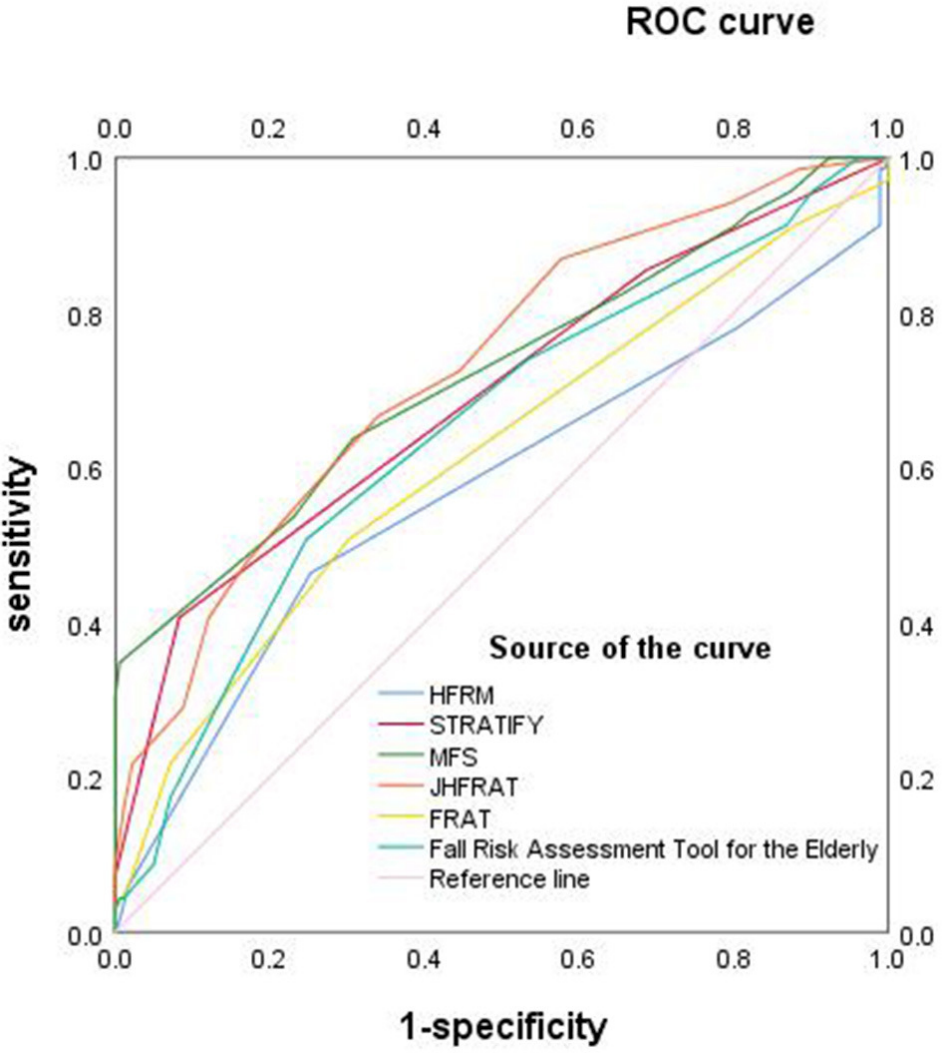
ROC curves of the six fall risk assessment scales.

Patient risk scores were calculated using the Hendrich Fall Risk Assessment Scale, Thomas Fall Risk Assessment Tool, Morse Fall Assessment Scale, Johns Hopkins Fall Risk Assessment Scale, Falls Risk Assessment Tool (FRAT), and the Fall Risk Assessment Tool for older adults. ROC curves were then plotted and used to assess the validity of different fall risk assessment scales. The AUC value was higher for the Johns Hopkins Fall Risk Assessment Scale (0.73, 95% CI: 0.66–0.80, *P* < 0.001) than the Morse Fall Assessment Scale (0.72, 95% CI 0.64–0.80, *P* < 0.001), Thomas Fall Risk Assessment Tool (0.69, 95% CI 0.61–0.77, *P* < 0.001), Falls Risk Assessment Tool for older adults (0.65, 95% CI 0.57–0.73, *P* < 0.001), Falls Risk Assessment Tool (FRAT) (0.62, 95% CI 0.53–0.70, *P* = 0.005), and the Hendrich Falls Risk Assessment Scale (0.57, 95% CI 0.49–0.66, *P* = 0.076) (Figure 1). The sensitivity and specificity of the Johns Hopkins Fall Risk Rating Scale, Morse Fall Assessment Older Scale, Thomas Fall Risk Assessment Tool, Falls Risk Assessment Tool for older adults, Falls Risk Assessment Tool (FRAT), and Hendrich Fall Risk Assessment Scale were 66.70%/65.90%, 34.80%/99.50%, 40.60%/91.80%, 50.70%/75.30%, 50.70%/69.80%, and 46.40%/74.70%, respectively. Of the six risk prediction models, the Johns Hopkins Fall Risk Assessment Scale had higher sensitivity and specificity for predicting fall risk among patients with hematological diseases, with 4.5 being the best cutoff point (Table 4).

**Table 4:** Comparison of the six different fall risk assessment scales in predicting the occurrence risk of falling in hospitalized patients with hematological diseases.

**Scales**	**AUC**	**Sensitivity (%)**	**Specifcity (%)**	**Youden index (%)**
HFRM	0.49–0.66	46.40	74.70	21.10
STRATIFY	0.61–0.77	40.60	91.80	32.40
MFS	0.64–0.80	34.80	99.50	34.30
JHFRAT	0.66–0.80	66.70	65.90	32.60
FRAT	0.53–0.70	50.70	69.80	20.50
Falls risk assessment tool for older adults	0.57–0.73	50.70	75.30	26.00

## 4. Discussion

Recent fall-related studies have focused on the epidemiology of older populations at home, in the community, or in apartments, and have largely assessed harm from falls resulting from the environment, osteoporosis, and diabetes (15). There has been a lack of research on the risk of falls among patients with hematologic diseases, despite the high incidence and serious consequences of falls in this patient population.

Diabetes, hypnotic drugs, the placement of catheters, hypoproteinemia, and anemia were identified as possible fall-related risk factors among patients with hematological diseases. This finding was consistent with a study by Miwa et al. (30). While Yanyan et al. found that falls may be associated with hemoglobin levels and age (31), this finding was not supported by this study. These differences may be related to longer bed rest, higher availability of family accompaniment, and a higher level of nursing care observed in the current study population. This study also found that fall-related risk factors were associated with disease stage and the intensity of chemotherapy, which account for some of the limitations in the Miwa et al. study. While univariate analysis identified several possible risk factors, multivariate analysis found that maintaining a stable disease stage, more intense chemotherapy regimens, and the use of sedatives, sleeping drugs, and diuretics were independent risk factors for falls, and the presence of a chaperone, a high level of nursing care, and high self-care scores were protective factors. The predictive ability of chemotherapy intensity, the use of diuretics, sedatives, and sleeping drugs, self-care ability, family accompaniment, and the level of nursing care used to avoid falls have been demonstrated by several clinical studies (11, 32–34). Interestingly, however, the hematological patients in our study who had stable-stage disease were more prone to falls. Better mobility, insufficient self-assessment, and less nursing care may account for this result. These findings highlight the importance of paying close clinical attention to hematologic patients who are being treated with sedative and diuretic medications, receiving intensive chemotherapy, and have stable-stage disease. In addition, the results suggest that falls may be reduced by increasing chaperone availability, improving nursing and family care, and improving patient self-care.

There are no clinically accepted fall-specific risk prediction models for inpatients with hematologic disorders. The classical fall assessment scales include the Hendrich Fall Risk Assessment Scale, Thomas Fall Risk Assessment Tool, Morse Fall Assessment Scale, Johns Hopkins Fall Risk Rating Scale, Falls Risk Assessment Tool (FRAT), and Fall Risk Assessment Tool for older adults. These models were assessed in specific study populations, such as emergency department patients, surgical patients, psychiatric disease patients, those seen in departments of general medicine, geriatrics, and geriatric rehabilitation, and older adults. Few studies have used these models to predict fall risk among patients with hematological diseases. Different patient populations have their own characteristics and risk factors. The current study suggested that the Johns Hopkins Fall Risk Assessment Scale had a relatively better predictive value for falls among inpatients with hematologic diseases, with an area under the ROC curve of 0.73, a finding similar to that found by Yadan et al. (35). This research group assessed the predictive power of the Johns Hopkins Fall Risk Assessment Tool in a general inpatient setting and calculated an area under the ROC curve of 0.73. Unlike the Yadan et al. study, however, the current report compared and validated the ability of six different risk assessment scales to predict falls among inpatients with hematologic diseases. The Johns Hopkins Fall Risk Rating Scale was found to have strong sensitivity and specificity to predict falls in hematology patients.

The current study has some limitations. First, it was a retrospective study conducted at a single center. Due to the small number of patients and limits of the available data, this study was unable to analyze all possible risk factors reported in the literature, including length of chemotherapy, allogeneic hematopoietic stem cell transplantation, patient and family awareness, limb dysfunction, and blood lipid level. Prospective studies with an expanded sample size are required to investigate other potential risk factors. Second, for ethical reasons, once patients were identified as being at high risk for falls, particular preventive measures were taken to ensure the safety of the study subjects, potentially leading to an underestimate of the fall incidence. Additional studies are needed to validate the findings in a larger population by increasing the frequency of assessment and exploring the use of more specific criteria to identify fall risk factors among inpatients with hematologic diseases. A quantitative assessment scale for monitoring fall-related risk factors could then be developed to effectively prevent them. This would provide the information needed to develop more practical prevention measures.

In conclusion, the use of sedative and diuretic drugs, intensive chemotherapy, and the maintenance of stable disease stages are independent fall-related risk factors among hospitalized patients with hematological diseases. For these patients, a more accurate assessment of self-care ability and enough care support from medical workers or families are necessary. A higher Barthel Index Score, a higher level of nursing care, and the availability of family accompaniment are protective factors to avoid falls among hematology patients. The Johns Hopkins Fall Risk Scale has a better predictive value for falls among hematology inpatients and can be used as a valid tool to predict fall risk in this population.

## Data availability statement

The original contributions presented in the study are included in the article/supplementary material, further inquiries can be directed to the corresponding author.

## Ethics statement

The studies involving human participants were reviewed and approved by Mianyang Central Hospital (Mianyang, China; approval no. S-2022-0224-01). The patients/participants provided their written informed consent to participate in this study.

## Author contributions

JW developed the study with FX. BC supervised the project. JW drafted the manuscript. FX revised the manuscript. All authors contributed to data collection and analysis. All listed authors approved the final manuscript.
